# Extended Polysaccharide Analysis within the Liposomal Encapsulation of Polysaccharides System

**DOI:** 10.3390/ma13153320

**Published:** 2020-07-26

**Authors:** Roozbeh Nayerhoda, Dongwon Park, Charles Jones, Elsa N. Bou Ghanem, Blaine A. Pfeifer

**Affiliations:** 1 Department of Biomedical Engineering, University at Buffalo, The State University of New York, Buffalo, NY 14260, USA; roozbehn@buffalo.edu; 2 Department of Chemical and Biological Engineering, University at Buffalo, The State University of New York, Buffalo, NY 14260, USA; dongwonp@buffalo.edu; 3 Abcombi Biosciences Inc., 1576 Sweet Home Road, Amherst, NY 14260, USA; charles.jones@abcombibio.com; 4 Department of Microbiology and Immunology, University at Buffalo, The State University of New York, Buffalo, NY 14260, USA; elsaboug@buffalo.edu

**Keywords:** liposome, serotype, polysaccharide, *Streptococcus pneumoniae*, vaccine, pneumococcal disease

## Abstract

The Liposomal Encapsulation of Polysaccharides (LEPS) dual antigen vaccine carrier system was assessed across two distinct polysaccharides for encapsulation efficiency, subsequent liposomal surface adornment with protein, adjuvant addition, and size and charge metrics. The polysaccharides derive from two different serotypes of *Streptococcus pneumoniae* and have traditionally served as the active ingredients of vaccines against pneumococcal disease. The LEPS system was designed to mimic glycoconjugate vaccines that covalently couple polysaccharides to protein carriers; however, the LEPS system uses a noncovalent co-localization mechanism through protein liposomal surface attachment. In an effort to more thoroughly characterize the LEPS system across individual vaccine components and thus support broader future utility, polysaccharides from *S. pneumoniae* serotypes 3 and 4 were systematically compared within the LEPS framework both pre- and post-surface protein attachment. For both polysaccharides, ≥85% encapsulation efficiency was achieved prior to protein surface attachment. Upon protein attachment with either a model protein (GFP) or a pneumococcal disease antigen (PncO), polysaccharide encapsulation was maintained at ≥61% encapsulation efficiency. Final LEPS carriers were also evaluated with and without alum as an included adjuvant, with encapsulation efficiency maintained at ≥30%, while protein surface attachment efficiency was maintained at ≥~50%. Finally, similar trends and distributions were observed across the different polysaccharides when assessed for liposomal zeta potential and size.

## 1. Introduction

Pneumococcal disease results from the virulent transition of *Streptococcus pneumoniae* bacteria that otherwise reside asymptomatically within the nasopharynx of a human host [1,2]. Pathological results include middle ear infections, pneumonia, and sepsis [3,4]. In addition to traditional therapy using antibiotics, vaccines have been developed based upon the polysaccharide capsule associated with the *S. pneumoniae* bacteria [5,6,7]. Complicating success, however, is the variation in capsule polysaccharide content resulting in so-called bacterial serotypes with slight variation in polysaccharide structure [8,9].

In the case of *S. pneumoniae*, there are >90 different bacterial serotypes which greatly complicate efforts to develop a universally effective vaccine candidate [10,11]. Namely, a vaccine based upon the surface polysaccharide immunogen would have to incorporate polysaccharides from all serotypes to be broadly effective, a task which is both scientifically and economically challenging [12].

Currently effective vaccine options include glycoconjugate brands that feature polysaccharide covalently coupled to a carrier protein [13]. In so doing, the resulting immune response is recognized as more potent and longer-lasting [12]. To date, vaccines of this type include ≥13 serotype polysaccharides per formulation [13,14]. However, extending vaccine coverage toward the remaining 90+ serotypes remains a daunting prospect.

**Figure 1:**
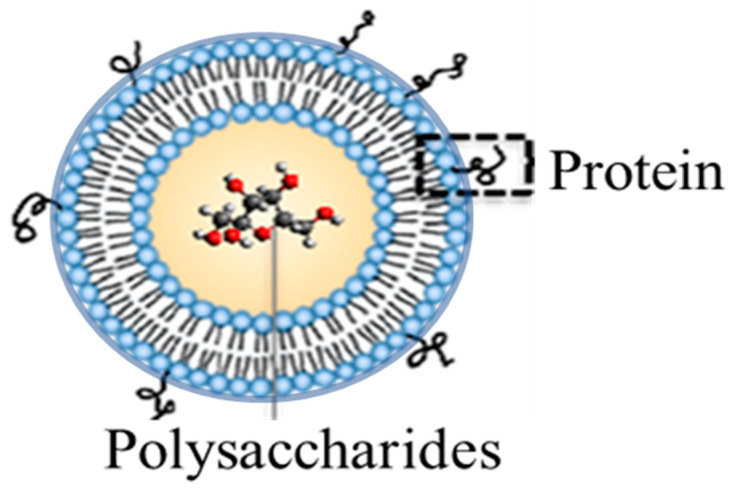
Liposomal Encapsulation of Polysaccharides (LEPS) system featuring polysaccharide immunogen content within the liposomal structure and proteins (either immune-stimulating or antigenic) noncovalently affixed to the outer surface via mechanisms that include metal-chelation (as in the case of the enclosed study).

In response, our research has focused on an alternative vaccine platform termed Liposomal Encapsulation of Polysaccharides (LEPS), in which a liposomal carrier simultaneously encapsulates polysaccharide content while allowing for the surface attachment of protein content (Figure 1) [8,12,15,16]. The end result is a glycoconjugate mimic that offers a simpler, noncovalent form of polysaccharide–protein co-localization and a more tractable and scalable route to full serotype vaccine coverage. In the current study, we characterize key steps in the LEPS construction process across multiple serotype polysaccharides and surface-attached proteins. Consistent product outcomes support the platform technology as being suitable for extended utility across the remaining serotype polysaccharide immunogens comprising a universally broad vaccine candidate.

## 2. Materials and Methods

### 2.1. Materials and Reagents

Liposomal construction material was obtained from Avanti Polar Lipids (Alabaster, AL, USA), ThermoFisher Scientific (Waltham, MA, USA), or Sigma Aldrich (St. Louis, MO, USA). Pneumococcal capsular polysaccharides (serotypes 19F, 4, and 3) were obtained from American Type Culture Collection (ATCC), and certain molecular details of the polysaccharides have been reported previously [17,18]. Green fluorescent protein (GFP) and a virulent-specific pneumococcal disease protein antigen (PncO) were produced recombinantly as previously reported [8,12,15,19]; briefly, the GFP and PncO proteins were generated through gene expression within *Escherichia coli*, with the protein products containing 6× histidine tags to facilitate affinity chromatography purification using a Ni-NTA packed bed column matrix.

### 2.2. Liposomal Preparation

The following 3:3:1:0.1:4 molar ratio of 1,2-dioleoyl-sn-glycero-3-phospho-(1’-rac-glycerol) (DOPG), 1,2-dioleoyl-sn-glycero-3-phosphocholine (DOPC), 1,2-dioleoyl-sn-glycero-3-[(N-(5-amino-1-carboxypentyl)iminodiacetic acid)succinyl] (nickel salt) (DGS-NTA(Ni)), 1,2-distearoyl-sn-glycero-3-phosphoethanolamine-N-[amino(polyethylene glycol)-2000] (ammonium salt) (DSPE-PEG(2000)), and cholesterol were dissolved in chloroform for liposomal formulations. To separate lipid mixtures, 1 mL of 0.2, 0.4, 0.6, 0.8, 0.85, and 1 mg/mL polysaccharide 4; 0.2, 0.4, 0.6, 0.7, and 0.8 mg/mL polysaccharide 3; and 0.6 mg/mL polysaccharide 19F were added, vortexed for 1 min, and evaporated using a rotatory evaporator to form a thin film, followed by 1× phosphate-buffered saline (PBS) rehydration at 45 °C using a rotatory evaporator rotated until the thin film was again fully dissolved. Samples were then passed 10 to 12 times through a handheld extruder with a 200 nm pore size membrane.

In order to separate liposomes encapsulating polysaccharide from free polysaccharide, 500 µL of post extrusion sample was transferred to a 300 kDa centrifugal tube (Pall Co., Post Washington, NY, USA) and centrifuged for 5 min at 4 °C and 1200 rcf. The resulting filtered sample volume was adjusted to the initial volume using PBS (with samples assessed at this stage for formulation metrics prior to protein surface attachment) and then subjected to protein binding by incubation with either GFP or PncO (280 µg) for 30 min at room temperature. To separate unbound protein from protein-bound liposomes encapsulating polysaccharides, samples were subjected to centrifugation purification, and the resulting liposomes were further purified with an additional centrifugation step. To assess the impact on LEPS construction metrics upon the addition of aluminum adjuvant (alum), 125 µg of aluminum phosphate was added to samples prior to an additional centrifugation purification step.

### 2.3. Polysaccharide Encapsulation Analysis

Liposome samples (0.6 mL) were mixed with 300 µL of 5% (w/v) phenol and 1.5 mL of concentrated sulfuric acid and vortexed for 5 s. The resulting solution (250 µL) was transferred to a Falcon^®^ 96-well microplate (Waltham, MA, USA), covered, and incubated for 15 min in a 92 °C water bath, followed by incubation at room temperature for the same duration, allowing the well plates to cool prior to colorimetric analysis at the wavelength optimum for polysaccharide analysis (Figure S1) using a Synergy Multi-Mode Microplate Reader (BioTek Instruments Inc., Winooski, VT, USA). Encapsulated polysaccharide values were calculated by comparison to a standard calibration curve and then divided by the initial amount of polysaccharide introduced to the liposomal production process to calculate % encapsulation efficiency values, with additional quantification details previously published by our group [20].

### 2.4. Size and Zeta Potential Analysis

After diluting samples in PBS, dynamic light scattering measurements were made using a Zetasizer Nano ZS90 instrument (Malvern, UK) to determine particle diameter and zeta potential of liposomes at 25 °C with a 4-mW, 633-nm HeNe laser as the light source at a fixed measuring angle of 90° to the incident laser beam.

### 2.5. Liposomal Protein Surface Assessment

Liposomal samples were assessed for GFP surface binding efficiency via fluorescence analysis at an excitation wavelength of 359 nm and an emission wavelength of 508 nm. When assessing PncO surface attachment, 50 µL of each standard (Bovine Serum Albumin Standard from Thermo Fisher Scientific, Waltham, MA, USA) or an experimental sample were placed into microplate wells prior to the addition of 300 µL of Pierce^TM^ Detergent Compatible Bradford Assay Reagent to each well, then pipetted up and down 4–5 times to mix the sample with the reagent. After incubation for 10 min at room temperature, samples were measured for absorbance at 595 nm (while subtracting blank measurements). Measurements were made using a Synergy 4 Multi-Mode Microplate Reader (BioTek Instruments Inc., Winooski, VT, USA). Resulting values were compared to standard protein calibration curves and divided by the initial amount of protein introduced to the liposomal surface binding step to calculate % efficiency values.

### 2.6. Experimental Repetition

Error bars associated with data represent results from three different experimental efforts.

## 3. Results

### 3.1. Liposomal Encapsulation of Streptococcus pneumoniae Serotype Capsular Polysaccharides 3 and 4

In previous work, we provided an in depth analysis of the LEPS formulation process, focused upon the encapsulation of capsular polysaccharide from *S. pneumoniae* serotype 19F [20]. In the current work, we sought to assess the consistency of the LEPS formulation process across alternative serotype polysaccharides, with positive results further supporting the LEPS platform as a potential universal vaccine system for pneumococcal disease. Thus, we first conducted liposomal encapsulation of polysaccharides from serotypes 3 and 4, which are both represented within clinically available glycoconjugate vaccines (as is serotype 19F) [13].

**Figure 2:**
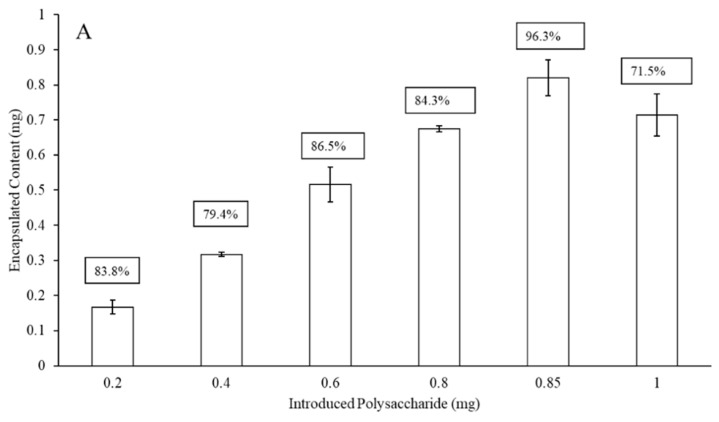

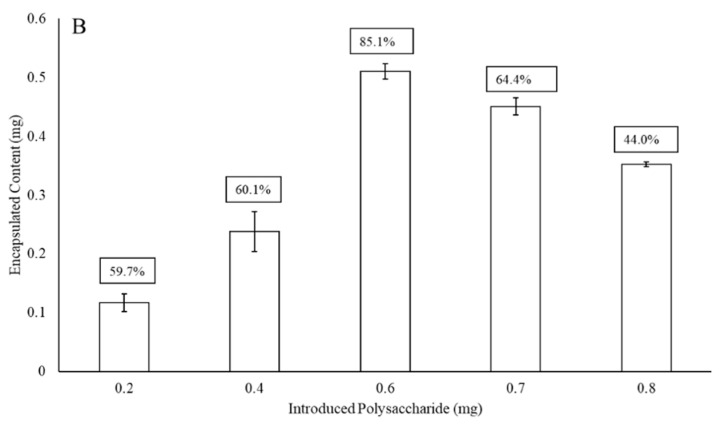
Liposomal polysaccharide encapsulation efficiency (boxed values; without surface protein addition) of serotype 4 (**A**) and 3 (**B**) as a function of initial polysaccharide introduced to the LEPS formulation process.

Figure 2 presents the maximum liposomal encapsulation efficiency for the serotype 3 and 4 polysaccharides (without protein surface binding) as a function of the initial amount of material introduced to the LEPS formulation process. In both cases, there is a maximum achieved across the range of 0.2 to 1 mg of initial polysaccharide introduced. The amount of polysaccharide introduced leading to the optimized encapsulation efficiency for the liposomal process then became the basis for all subsequent formulations (and this same optimal input amount was used in the case of serotype 19F polysaccharide in data presented later). The maximum encapsulation efficiencies for serotype 3 and 4 polysaccharides were 85% and 96%, respectively.

### 3.2. LEPS Surface Protein Binding Impact upon Polysaccharide Encapsulation Efficiency

The LEPS platform offers the noncovalent attachment of protein to the surface of the liposomal carrier to mimic the glycoconjugate vaccines that have been used effectively as pneumococcal disease prophylactics [12]. For the LEPS system, protein attachment is completed through metal coordination chemistry in which nickel-doped liposomal content chelates 6× histidine-tagged recombinant protein products (initially, green fluorescent protein (GFP)) [8,20]. The data presented in Figure 2 support efficient polysaccharide encapsulation within the liposomal formulation prior to surface protein attachment. The next step in analysis would then be to fully functionalize the LEPS particle with a surface-localized protein. Thus, to extend upon our liposomal encapsulation studies for polysaccharides 3 and 4, we assessed the impact protein surface attachment had upon encapsulation efficiency.

**Figure 3:**
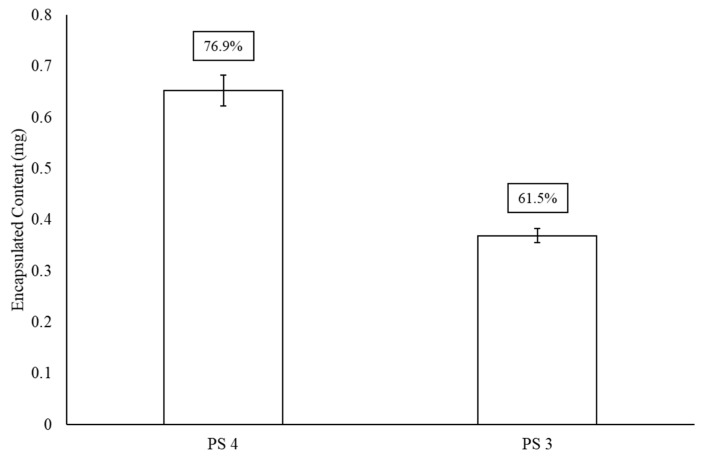
LEPS encapsulation efficiency (boxed values) of polysaccharides (PS) 4 and 3 after GFP protein surface binding.

Figure 3 presents encapsulation efficiency of polysaccharides 3 and 4 after GFP has been noncovalently affixed to the surface of the liposome. This particular protein was chosen for its model quantification properties. When comparing Figure 2 and Figure 3, there is an approximately 20–25% drop in LEPS encapsulation efficiency for both polysaccharides 3 (0.6 mg introduced polysaccharide sample) and 4 (0.85 mg introduced polysaccharide sample), resulting in final levels of ~62% and ~77% (a combined 24% reduction in average encapsulation efficiency), respectively.

### 3.3. The Addition of Adjuvant upon LEPS Formulation Parameters

Adjuvants are a common additive to vaccines designed to boost overall immunogenicity and the effectiveness of the final immune response [21,22,23]. Alum is a common adjuvant and one we have used previously in LEPS formulations for pneumococcal disease [8,12]. As such, we sought to measure the effect the alum adjuvant has upon LEPS formulation parameters.

**Figure 4:**
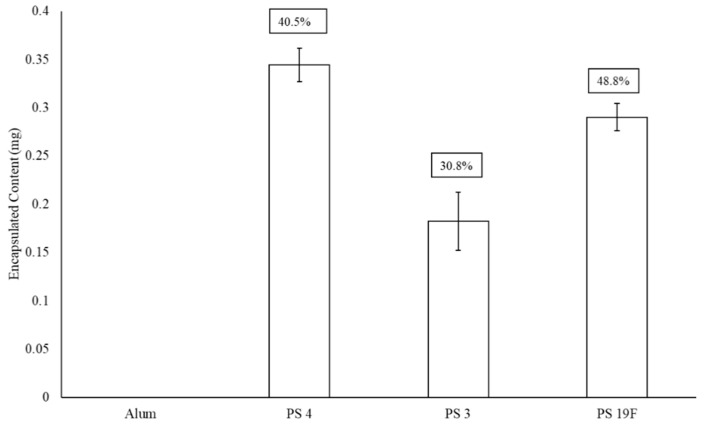
Effect of adjuvant (alum) on LEPS polysaccharide (PS) encapsulation efficiency (boxed values, with comparison to best values for PS 4 and 3 in Figure 2 and Figure 3; with GFP surface protein addition). Alum was tested with a liposomal sample without PS to confirm lack of background signal interference.

Figure 4 presents the impact alum has upon final LEPS polysaccharide encapsulation. Here, there is another drop in encapsulation efficiency (30–36%) from that previously observed upon GFP surface attachment (a combined 61% reduction in average encapsulation efficiency relative to Figure 2). Included in this analysis is the 19F polysaccharide encapsulation efficiency, where a drop in encapsulation efficiency of ~13% is observed relative to a previous analysis without alum addition [20].

**Figure 5:**
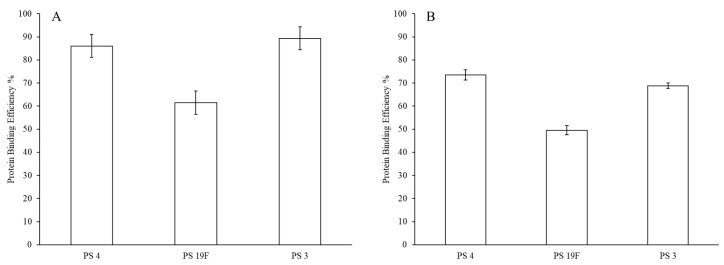
Green fluorescent protein (GFP) LEPS surface attachment efficiency without (**A**) and with (**B**) alum.

GFP protein surface attachment efficiency for the final LEPS particles is presented with and without alum addition in Figure 5. When compared to the polysaccharide encapsulation efficiency data in Figure 5, alum has less of a pronounced effect on protein surface binding efficiency, though there is still a drop of ~10–15%. In this analysis, we also observe a difference in overall GFP surface binding across encapsulated polysaccharides. Without alum, strong surface binding efficiency values of ~85–90% are observed for polysaccharides 3 and 4, whereas protein binding efficiency is ~60% for polysaccharide 19F.

**Figure 6:**
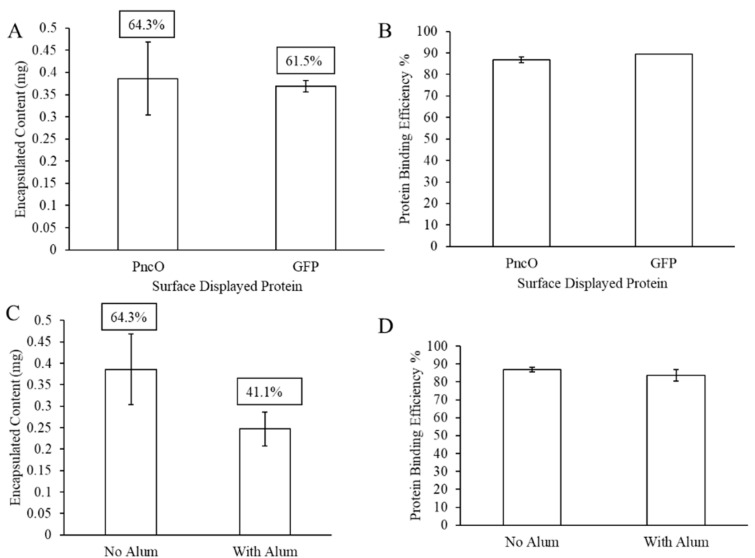
LEPS formulation with a virulent-specific pneumococcal disease protein antigen (PncO) surface protein attachment. Assessment of polysaccharide 3 encapsulation and surface protein binding efficiency compared to GFP (**A**,**B**) and with alum (**C**,**D**).

The results thus far presented prompted a second evaluation, summarized in Figure 6, using a protein, PncO, previously identified as an antigen for pneumococcal disease [8,12,15,19]. In particular, PncO was indicated as a biomarker associated with virulent *S. pneumoniae* cells, thus serving as a good antigen candidate in LEPS vaccine formulations [15,16,24,25,26]. Fixing the polysaccharide content of the LEPS formulation to that of serotype 3, encapsulation efficiency was compared across subsequent protein surface attachment using either GFP or PncO, with encapsulation retained at levels ≥61%. Surface protein binding levels were nearly identical when comparing the GFP and PncO LEPS formulations. Finally, when LEPS formulations with PncO included alum, polysaccharide encapsulation efficiency dropped ~23% (in a trend similar to that observed when using GFP as the surface protein), though protein binding efficiency was only minimally affected.

**Figure 7:**
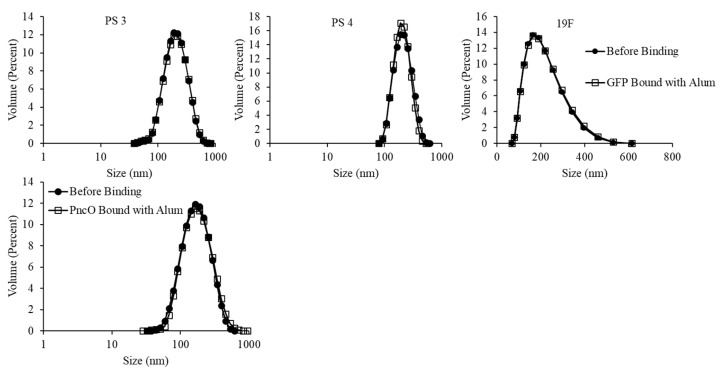
Size distribution analysis of LEPS particles with and without GFP/PncO/alum addition. Data presented with PncO utilized polysaccharide (PS) 3.

Figure 7 and Table 1 present liposomal size distribution and zeta potential values, respectively. Liposomal size distributions remain normalized upon protein surface binding and with the addition of alum, though there is a generally broader peak around a larger average size for the liposomal sample encapsulating polysaccharide 19F. The zeta potential values in Table 1 show a generally consistent trend of negative surface charge across samples with and without alum.

## 4. Discussion

The LEPS platform provides an alternative formulation to the glycoconjugate vaccine options developed previously for pneumococcal disease [8]. The advantage of the glycoconjugates is the enhanced immune response, featuring antibody class switching and extended memory, relative to vaccines reliant only upon polysaccharide immunogens [12,16]. The LEPS particle mimics this conjugate feature, though through noncovalent mechanisms, via the proximal localization of polysaccharide and protein within and on the liposomal vehicle, respectively [8,20,27].

A challenge to broad utility of the current glycoconjugate vaccines is the limited number of polysaccharides that can be economically included within final formulations (as each glycoconjugate requires dedicated processes to produce, purify, and quality control both the protein and polysaccharide components and the final glycoconjugate product) [9,28]. The LEPS vehicle has the potential to alleviate this production challenge through either the individual or combined encapsulation of polysaccharides together with a simple, noncovalent protein attachment mechanism.

Thus, a focus of the enclosed work was a deeper assessment of the LEPS formulation process across multiple polysaccharide components, from *S. pneumoniae* serotypes 3 and 4, found within current glycoconjugate vaccine products. Generally, the LEPS formulation process showed consistency across the 19F, 3, and 4 serotype polysaccharides that were evaluated in this study more fully. Differences in encapsulation efficiency, in particular, were observed more noticeably upon protein surface attachment and adjuvant addition. Such downstream formulation steps likely influenced the structural integrity of the liposomal construct, as we also observed previously [20] (as opposed to the more passive encapsulation of polysaccharide within newly formed liposomes), thus exacerbating differences observed upon initial polysaccharide liposomal encapsulation. Variation in liposomal encapsulation likely also reflects inherent differences in polysaccharide molecular weight and chemical structure, noting that similar variation in polymeric macromolecules can have a strong influence on vaccine delivery vehicles [29,30]. Though liposomal size and Zeta potential remain relatively stable, variations observed (especially for surface charge) are likely correlated to protein/alum addition and the corresponding impact upon polysaccharide encapsulation. Regardless, the final encapsulation values would dictate the amount of LEPS needed during a typical vaccination attempt, which thus argues for continual upgrades to the formulation process to boost overall polysaccharide/protein co-localization efficiency. A future avenue to do so would include a separate systematic variation of the foundational liposomal lipids, as a preface to better initial and/or retained polysaccharide encapsulation.

LEPS protein surface attachment efficiency was consistently strong (>60%) across the polysaccharide samples tested in this study. However, the addition of alum again lowered this degree of efficiency. Adjuvants like alum have become key ingredients of vaccines, including prior efforts with liposomal carriers [8,12,15], due to their positive impact upon overall immunogenicity and final prophylactic responses [31]. However, in future studies, we intend to evaluate the LEPS formulation with and without additional alum adjuvant. A similar degree of immune response success in such comparisons would then support the exclusion of alum from final LEPS formulations. This would be a positive development for two reasons: (1) The removal of alum would lead to both a simpler and more cost-effective final vaccine, and (2) improved polysaccharide encapsulation and surface binding efficiency values without alum addition would thus save on these raw materials as well. More broadly, a LEPS formulation able to be effective without external alum would improve overall production efficiency, leading to broader utilization, due to a reduced cost barrier.

## 5. Conclusions

The LEPS system was analyzed across multiple *S. pneumoniae* serotype polysaccharides for encapsulation efficiency with and without both protein (GFP, PncO) surface binding and adjuvant (alum) addition. Initial liposomal encapsulation efficiency of serotype 3 and 4 polysaccharides reached or exceeded 85%. Surface attachment of protein reduced polysaccharide 3 and 4 encapsulation ~20–25%, though protein surface attachment efficiency was ≥85%. The addition of external adjuvant (alum) to LEPS formulations had little effect on final particle size and charge characteristics, but reduced both encapsulated polysaccharide content (~30–36%) and surface GFP attachment (~10–15%), suggesting a formulation effective without the need for external adjuvant would result in antigen dose sparing and an overall more economical final vaccine product.

## Figures and Tables

**Table 1:** LEPS surface charge analysis.

LEPS Particle	Zeta Potential (mV)
Encapsulating PS 19F (Pre-protein Binding)	−12.6
Encapsulating PS 3 (Pre-protein Binding)	−28.7
Encapsulating PS 4 (Pre-protein Binding)	−30.7
Encapsulating PS 19F (Post-GFP Binding with Alum)	−17.3
Encapsulating PS 3 (Post-GFP Binding with Alum)	−26.7
Encapsulating PS 3 (Post-PncO Binding with Alum)	−8.5
Encapsulating PS 4 (Post-GFP Binding with Alum)	−12.6

## Supplementary Materials

The following are available online at https://www.mdpi.com/1996-1944/13/15/3320/s1, Figure S1: Absorbance spectrum for polysaccharides 4 and 3.
