# Supplementary material for: Extended Polysaccharide Analysis within the Liposomal Encapsulation of Polysaccharides System
Source: Materials (Basel). 2020 Jul 26;13(15):3320. doi: 10.3390/ma13153320 (PMC7436327; doi:10.3390/ma13153320)
Supplement: Supplementary file 1 [file materials-13-03320-s001.pdf]

Supplementary Materials

Extended Polysaccharide Analysis within the Liposomal Encapsulation of Polysaccharides System

Roozbeh Nayerhoda [1], Dongwon Park [2], Charles Jones [3], Elsa N. Bou Ghanem [4] and Blaine A. Pfeifer [1,2,*]

[1] Department of Biomedical Engineering, University at Buffalo, The State University of New York, Buffalo, NY 14260, USA; roozbehn@buffalo.edu
[2] Department of Chemical and Biological Engineering, University at Buffalo, The State University of New York, Buffalo, NY 14260, USA; dongwonp@buffalo.edu
[3] Abcombi Biosciences Inc., 1576 Sweet Home Road, Amherst, NY 14260, USA; charles.jones@abcombibio.com
[4] Department of Microbiology and Immunology, University at Buffalo, The State University of New York, Buffalo, NY 14260, USA; elsaboug@buffalo.edu
* Correspondence: blainepf@buffalo.edu

Received: 21 June 2020; Accepted: 23 July 2020; Published: 26 July 2020

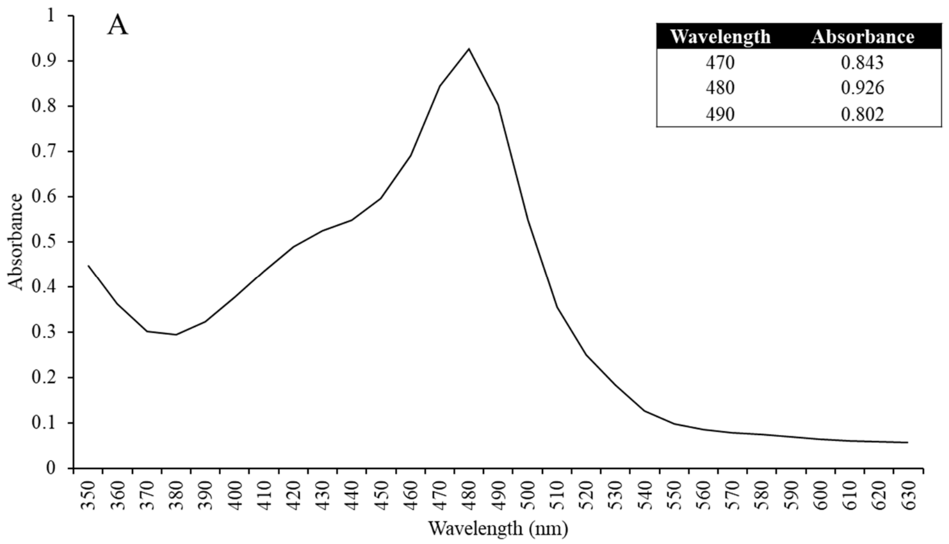

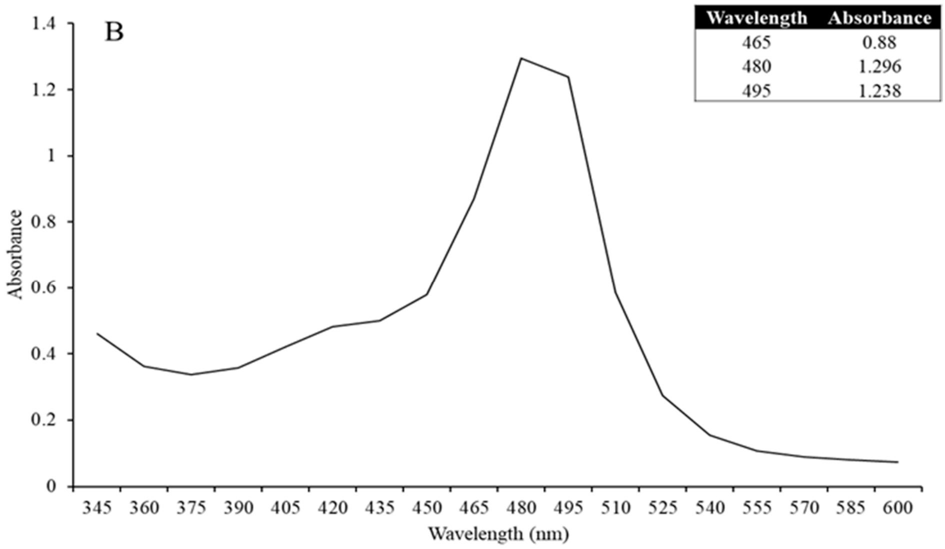

Figure 1. Absorbance maximum for polysaccharides 4 (**A**) and 3 (**B**).

© 2020 by the authors. Licensee MDPI, Basel, Switzerland. This article is an open access article distributed under the terms and conditions of the Creative Commons Attribution (CC BY) license (http://creativecommons.org/licenses/by/4.0/).
